# The Impact of the Parental Support on Risk Factors in the Process of Gender Affirmation of Transgender and Gender Diverse People

**DOI:** 10.3389/fpsyg.2018.00399

**Published:** 2018-03-27

**Authors:** Bruna L. Seibel, Bruno de Brito Silva, Anna M. V. Fontanari, Ramiro F. Catelan, Ana M. Bercht, Juliana L. Stucky, Diogo A. DeSousa, Elder Cerqueira-Santos, Henrique C. Nardi, Silvia H. Koller, Angelo B. Costa

**Affiliations:** ^1^Departamento de Psicologia, Faculdade Cesuca, Cachoeirinha, Brazil; ^2^Programa de Pós-Graduação em Psicologia, Universidade Federal do Rio Grande do Sul, Porto Alegre, Brazil; ^3^Programa de Identidade de Gênero (PROTIG), Hospital de Clinicas de Porto Alegre (HCPA), Universidade Federal do Rio Grande do Sul, Porto Alegre, Brazil; ^4^Programa de Pós-Graduação em Psicologia, Pontifícia Universidade Católica do Rio Grande do Sul, Porto Alegre, Brazil; ^5^Laboratório de Avaliação e Medição em Psicologia, Universidade Tiradentes, Aracaju, Brazil; ^6^Programa de Pós-Graduação em Psicologia, Universidade Federal de Sergipe, São Cristóvão, Brazil; ^7^Programa de Pós-Graduação em Psicologia Social, Universidade Federal do Rio Grande do Sul, Porto Alegre, Brazil; ^8^Optentia Research Focus Area, North-West University, Vanderbijlpark, South Africa

**Keywords:** parental support, gender affirmation, self-esteem, homelessness, transgender, gender diversity

## Abstract

Research involving transgender and gender diverse people (TGD) increased in the last years, mostly concerning healthcare associated to this population. Few studies dedicated their analysis to the impact of parental support on transgender people, even though this is an important aspect in creating a safe environment on which these individuals can build their identity. In addition, the link between family support, TGD identity and homelessness is not completely established. Thus, due to the specificities of the family context of TGD individuals, the aim of this study is to investigate the association between family support and TGD in different moments of the process of gender affirmation. In addition, this study also aims to explore the relationship between a lack of social support and low self-esteem, home abandonment, and dwelling in the street. The survey was designed based on the TransPULSE project and was made available in electronic format. The sample was constituted of 423 TGD residents in two Brazilian states. A Structural Equation Model analysis suggested that the impact of gender affirmation status on homelessness was mediated by parental support, through self-esteem, and the need to move from home. The association between the status of the gender affirmation procedures, family support and self-esteem was significant and indicated that the further TGD individuals advanced in gender affirmation, the more self-esteem and family support they would have. The association between family support and self-esteem indicated that family support was associated with higher self-esteem. Low family support was associated with the willingness to move from home due to one’s TGD status and there was also a significant correlation between low self-esteem and the willingness to move from home due to one’s TGD status. Finally, homelessness was associated with the willingness to move with a large effect size. Limitations include the sample that was constituted by individuals with Internet access and who had more contact with TGD communities. The findings indicate directions for interventions involving TGD people and their families, considering the parental relationship as a critical variable to improve TGD quality of life in the process of gender affirmation.

## Introduction

Transgender and gender diverse (TGD) is an umbrella term that encompasses a wide range of individuals who do not identify with the gender assigned to them at birth, as opposed to cisgender individuals, who identify with the gender assigned to them (Ansara and Hegarty, 2012). TGD individuals are systematically vulnerable to grave rights violations. Stigma, prejudice and violence are common in their trajectories, and these individuals constantly experience labor discrimination (Clements-Nolle et al., 2006), lack of support (Soares et al., 2011), unemployment (Divan et al., 2017), high rates of depressive symptoms and suicide (Bockting et al., 2013), and precarious access to health services, among other difficulties (American Psychological Association [APA], 2015).

When referring to TGD individuals, many researchers work with the idea of a weakened social support network marked by stigma and prejudice toward individuals who are part of this group. Even with the progress made in the medical and surgical fields and in psychotherapy, the support and sustenance from the social relationships that are important to TGD individuals are not always comprehensive or satisfactory (Zamboni, 2006; Silva and Cerqueira-Santos, 2014).

The expectations of the social support network include serving as company and support and as a potential source of material and service resources. In addition, it is in these relationships with other individuals, such as family, close friends or more formal relationships, such as with professional colleagues, that TGD individuals can build their identities (Grant et al., 2011; Soares et al., 2011).

Given this wide range of groups and individuals who can provide social support, one of the major sources of support comes from the family environment. The family environment is where initial bonds are formed, in which the individual learns to relate to the world and develop linkages of affection (Costa and Dell’Aglio, 2009). Therefore, parents have a crucial role in promoting well-being (Resnick et al., 1997). In fact, family acceptance has been associated with young adult positive health outcomes (such as self-esteem) (Ryan et al., 2010). Furthermore, high levels of support in the family group are an important component of the process of gender affirmation (Winck and Petersen, 2005) and it seems to enhance after gender confirmation surgery (GCS), suggesting greater acceptance of the TGD identity by the family. In contrast, familial maltreatment is frequently linked with homelessness (Choi et al., 2015), as well as negative health outcomes (such as depression, substance abuse, and suicidal ideation and attempts) (Ryan et al., 2010).

In spite of the exponential increase in studies examining TGD issues, very few studies have been conducted on families of TGD. An analysis of the literature on TGD individuals revealed that few articles have addressed the experiences of family members, friends, and romantic partners of TGD individuals (Erich et al., 2008; Ryan et al., 2010; Stotzer, 2011; Snapp et al., 2015).

Despite not being included in most studies, the families and friends of TGD individuals are affected by their TGD identity in a variety of ways. This group has been colloquially named Significant Others, Family, Friends, and Allies Resources (SOFFA). SOFFA is engaged in the complex decision-making process of gender affirmation and the social changes it evolves. Their views on gender and relationships will be redefined. These processes influence the relationships between the TGD individual and each family member (Zamboni, 2006). Clearly, there is a lack of literature on SOFFAs (Ellis and Eriksen, 2002; Zamboni, 2006), especially considering that they may suffer transphobia in social settings (Cook-Daniels, 2001), as well as face anxiety not only for TGD person’s identity transition but also their own (Ellis and Eriksen, 2002; Zamboni, 2006).

Reactions of support from SOFFA with regard to TGD issues may oscillate between stages (Emerson and Rosenfeld, 1996). The first stage includes embracing the feelings of shock, betrayal, and confusion that a family may express after the disclosure. Similarly, the second stage describes the stress and conflict a family can experience upon knowing the reality of gender variance, which can provoke different reactions. The third stage is a negotiation step, in which the family discusses these challenges, begins to accept the reality of a gender variant relative and attempts to soften their functioning rules. The last stage, finding balance, describes a period in which secrets are eliminated, and the family members no longer experience turmoil or the need to negotiate differences, which does not necessarily signify a resolution (Lev, 2004).

Between 30 and 40% of homeless youth are lesbian, gay, bisexual, TGD, queer, or questioning (LGBTQ) individuals. According to the 2014 LGBTQ Homeless Youth Provider Survey that evaluated 138 youth homelessness human service agency providers, the proportion of LGBTQ youth these agencies served over the past 10 years increased (Corliss et al., 2011). The link between family support, TGD identity and homelessness is not completely established. Thus, due to the specificities of the family context of TGD individuals, the aim of this study is to investigate the association between family support and TGD identity in different moments of the process of gender affirmation. In addition, this study also aims to explore the relationship between a lack of social support and low self-esteem, home abandonment, and dwelling in the street. The study is based on the perspective of minority stress i.e., that discrimination influence psychosocial outcomes in sexual and gender minorities (Hendricks and Testa, 2012).

## Materials and Methods

### Design

The “Trans Health Research Project” is a hospital and web-based cross-sectional survey that was built with input from the medical and TGD communities to assess the healthcare needs of and access barriers for transgender and gender diverse people (TGD) residents in two Brazilian states. It is an evidence-informed, policy-making initiative of the Universidade Federal do Rio Grande do Sul (UFRGS) in tandem with the Universidade de São Paulo (USP).

### Ethical Considerations

The project was submitted and approved by the Ethical Committee and Research Commission of Hospital de Clínicas de Porto Alegre. In addition, in the Ethical Committee of Hospital de Clínicas of Medicine School of Universidade de São Paulo and in the Research Commission of Ethical Committee of Universidade Federal do Rio Grande do Sul Psychology Institute. Participants were informed about the objectives and functioning of the research and a free and informed consent term was provided. Confidentiality and anonymity were assured; moreover, the participations were voluntary with the possibility to withdraw at any time. It was also informed the inexistence of direct benefits for participating in the survey, clarifying that it would not influence healthcare provision, in case of the participants that completed the questionnaire in hospitals.

### Data Collection

Data were collected in two Brazilian states that pioneered the provision of specialized services for TGD since the Brazilian health policies for TGD were first implemented in Rio Grande do Sul, Brazil’s southernmost state, and São Paulo, a state in the southeast region. Both states have gender identity programs that provide GCS at university hospitals. Since the Brazilian Unified Health System (SUS) provides georeferenced care, patients seeking gender affirmation must access those procedures in the states in which they live. Patients in the two programs were voluntary invited to complete an electronic version of the survey. The questionnaire was also available on the internet through an online Facebook announcement during two time periods: July–October 2014 and January–March 2015. The announcement was displayed for users of Facebook who indicated the following characteristics in their profiles: lived in the states of São Paulo and Rio Grande do Sul; were 18 years or older; and “liked” pages on Facebook and joined groups or events related to transsexuality, TGD individuals and the LGBT movement. Facebook statistics indicated that the ad had 521,601 impressions (number of times the ad was shown on the site), with 7,226 ad clicks. The click rate indicated that 0.72% of the impressions drove individuals to the main page of the study. IP number of computers was registered in order to withdraw participants that completed the survey more than once.

### Materials

The survey was modeled after the TransPULSE project. TransPULSE was one of the first large-scale studies that addressed the health needs and vulnerabilities of TGD and their barriers to healthcare access. The project aimed to improve the quality of life of TGD in Ontario, Canada by measuring levels of social exclusion and their impact on physical and mental health. The procedure for cross-cultural adaptation of the instrument for Brazilian populations was based on Borsa et al. (2012), according to the following steps: (1) contextual equivalence and review by expert committee; (2) translation; (3) evaluation by the target audience; and (4) evaluation by the original authors of the instrument. For this study, the survey was adapted for use in the Brazilian population by a group of health practitioners who worked in gender and sexuality diversity and who were assessed by Brazilian TGD community members.

The Brazilian survey was composed of 122 items grouped into 11 categories: 1- demographic characteristics; 2- motherhood/fatherhood; 3- physical health; 4- use of sexual hormones; 5- gender reaffirming procedures; 6- sexually transmitted infections and HIV; 7- experiences of discrimination in healthcare (violence experience, suicidal attempts), work and educational contexts; 8- dwelling; 9- income; 10- abusive use of alcohol and other drugs; and 11- sexual and mental health.

Gender identity was assessed using the two questions method (one related to the gender designated at birth and another asking how the participants describe their gender identity), and people were considered eligible for participation if they reported a gender different from that assigned to them at birth (Bauer et al., 2017). The organized Brazilian social movement prefers the use of the terms *travesti*, transsexual and trans person (man or woman) to the anglophone umbrella term transgender. *Travesti* is a culturally specific gender identity term for Brazilians who were designated as male at birth but affirm a female gender performance and bodily form, although they typically do not undergo neovaginoplasty. Their gender identity varies: most identify as male, some identify as women, and others simply identify as *travesti*. Based on their self-reported gender identity, participants were re-categorized as transgender women, transgender men or gender non-conforming people. Transgender women were those who were designated as male at birth but identified as women, transgender women or *travestis.* Transgender men were those who were designated as female at birth but identified as men or transgender men. Finally, gender non-conforming persons were those who identified with a gender identity outside the binaries (male–female), such as queer, non-binary, a-gender, etc.

The scale of support for transsexual identity was developed for the TransPULSE project (Bauer, 2012). The scale assesses the extent to which friends, family, and colleagues support the transsexual identity of the respondent. The answers were evaluated on a 4-point scale with the following response options: “they do not support at all,” “they do not support much,”, “they support a little,” “they strongly support,” and “not applicable.” The final score was determined by the number of answers indicating support: lower scores or scores up to 1 are classified as having very little or no support, scores between 1 and 2 are classified as having little support, scores between 2 and 3 are classified as having some support, and scores with values greater than 3 are classified as having strong support. Cronbach’s alpha value was 0.83.

Family support was calculated on a 3-point scale with the following response options: 1 or “no support,” 2 or “support a little,” and 3 or “support or support a lot.” In addition, participants could mark that they had no mother or father. Cronbach’s alpha for this sample considering the overall scale was 0.90. For this study, only support related from father/mother was considered. Furthermore, participants were asked the following question: “how often do you need to move away from your family or friends for being TGD?”. The answer choices were rated on a 5-point Likert scale ranging from 1 (never) to 5 (always). Finally, they were asked the following question: “do you already live without fixed housing?” If the respondent answered yes, they were asked about the context of their home.

The Brazilian version (Avanci et al., 2007) of the Rosenberg (1965) Self-Esteem Scale was used to evaluate the respondent’s self-esteem. This scale contains ten affirmations concerning high or low self-esteem rated on a 4-point Likert scale with the following response options: “I totally agree,” “I agree,” “I disagree,” and “I strongly disagree.” Higher scores on this scale indicate higher levels of self-esteem. Cronbach’s alpha for this sample was 0.86.

### Participants

Seven hundred and one TGD participants completed the survey, and 423 met the inclusion criteria for this study. The average age of the participants was 27.06 years (95% CI [26.22, 27.90 years]; *SD* = 8.74 years; median 24 years), and the age range of the participants was 18–61 years. Additional sociodemographic data are presented in **Table 1**.

**Table 1:** Participants sociodemographic data.

	*n* (%)
Gender identity (*n* = 423)	
Transgender men	265 (61.65)
Transgender women	124 (29.31)
Gender non-conforming persons	34 (8.04)
Medical gender affirmation status (hormone, surgery, silicone, etc.) (*n* = 421)	
Done	61 (14.49)
Doing	186 (44.18)
Plan to do	124 (29.45)
Not sure	32 (7.60)
Will not do	18 (4.28)
Age (*n* = 418)	
18–24	212 (50.72)
25–34	132 (31.58)
35–44	50 (11.96)
45–54	19 (4.55)
55–64	5 (1.20)
Race/color/ethnic (*n =* 533)	
White	313 (74.00)
Non-white	110 (26.00)
Parda	70 (16.55)
Yellow	12 (2.84)
Indigenous	2 (0.47)
Black	26 (6.15)
Education (*n* = 423)	
None	6 (1.42)
Fundamental education	40 (9.46)
Middle education	271 (64.07)
Higher education	81 (19.15)
Postgraduate studies	25 (5.91)
Population of city of residence (*n* = 423)	
5000–20000 inh	51 (12.06)
20000–50000 inh	38 (8.98)
50000–100000 inh	46 (10.87)
100000–500000 inh	108 (25.53)
More than 500000 inh	180 (42.55)

### Data Analysis

Central tendency and frequency statistics were calculated for the survey variables analyzed in this article. Relationships between outcomes and contextual variables were calculated using χ2 tests with the phi (φ) coefficient to determine the magnitude of the effects. The differences were considered to be significant when the *p*-value was smaller than 0.05, the 95% confidence interval did not include 0 and the effect size was larger than 0.15, which denote medium to strong effects of exogenous latent variables according to the general (Cohen, 1988).

Structural equation modeling (SEM) was used to investigate the fit of the theoretical model considering the associations between the stages of the gender affirmation procedure, the level of family support and self-esteem, the need to move out for being TGD and the occurrence of homelessness. The tested model included the following variables and associations: (1) steps on whether the process of gender affirmation would predict family support and a TGD individuals’ self-esteem; (2) whether a TGD individuals’ low self-esteem would correlate with their willingness to leave home and whether lack of family support would predict their willingness to leave home; and (3) whether willingness to leave home would predict homelessness. The correlation matrix and the Variance Inflation Factor (VIF) values among all variables in the model were calculated in order to test the required assumption that there was no multicollinearity impairing the SEM analyses.

## Results

### Parental Support for TGD Identity

Of the total of 421 respondents in relation to a parents’ support for one’s TGD identity, 29.45% (*n* = 124) of the sample answered that their parents did not at all support their status, 20.43% (*n* = 86) answered that their parents supported their status only a little, 20.43% (*n* = 86) answered that their parents supported them, 24.23% (*n* = 102) answered that their parents supported them completely, and 5.46% (*n* = 23) answered that they did not have a father and/or mother.

Welch’s adjusted F ratio was used in the one-way ANOVA, which demonstrated statistically significant differences between the groups: Welch’s *F*(2,82.62) = 9.06, *p* < 0.001, ω^2^ = 0.04. Additionally, the Games-Howell *post hoc* test indicated that gender-diverse persons had lower parental support than transgender women (Δ = -0.62, 95% CI [-0.99, -0.25], *p* = 0.001) and transgender men (Δ = -0.42, 95% CI [-0.81, -0.03], *p* < 0.05). Nevertheless, transgender men and transgender women did not differ from each other regarding parental support.

There was also a significant difference in parental support regarding the medical gender affirmation status *F*(3,394) = 4.83, *p* < 0.05, η^2^ = 0.04. Participants who had already completed the gender affirmation procedures did not differ from those who were in the process of completing them. However, those who had already performed the procedures had greater parental support than those who planned to complete the process (Δ = 0.47, 95% CI [0.11, 0.83], *p* < 0.05) and those who were in doubt or would not complete the process (Δ = 0.49, 95% CI [0.04, 0.93], *p* < 0.05). Finally, those who were in doubt or would not complete the process did not differ in support relative to those who did plan to complete the process (**Table 2**).

**Table 2:** Family support to TGD identity regarding medical gender affirmation status.

	Very little or no support *n* = 124 (%)	Little support *n* = 86 (%)	Some or strongly support *n* = 188 (%)
Done	11 (19.64)	8 (14.29)	37 (66.07)
Doing	49 (27.22)	42 (23.33)	89 (49.44)
Plan to do	47 (40.52)	23 (19.83)	46 (39.66)
Not sure	17 (36.96)	13 (28.26)	16 (34.78)

### Parental Support and Self-Esteem

Considering only those who did not receive any support (*M* = 14.54, *SD* = 6.05) versus those who received little support, some support and high levels of support (*M* = 18.75, *SD* = 6.07), there was a difference between the levels of self-esteem (*t*(354) = -6.11, *p* < 0.001 *d* = 0.69). Scores below 15 suggest low self-esteem. The mean score of non transgender participants in Brazil was 18.9 (*SD* = 6.78) (Hutz and Zanon, 2011).

### Parental Support and Housing

Of the total 410 participants who were asked if they needed to move away from their family because they were TGD, 164 (40.00%) answered yes. Besides, foram a total of 388 participantes who responded if they received any parental support, the majority (64.17%, *n* = 77) of participants who did not receive support needed to leave home, compared to 29.85% (*n* = 80) of participants who received some support. Family support significantly affected the need to leave home: χ2 (1, *N* = 388) = 40.52, *p* < 0.001, and φ = 0.32. The lack of family support increased the need to leave home by 4.21 times (odds ratio of 95% [2.67, 6.64]). In relation to these data, it is important to point out that some parts of the survey were optional, so the frequencies for each option varied as the total number of respondents for each question.

In addition, 13.00% (*n* = 13) of the participants answered that they were already without fixed housing, of a total of 416 respondents. Of those who did not receive any support from their parents, 23.57% (*n* = 29) stated that they did not have fixed housing, compared to 7.38% (*n* = 20) of those who already received some type of support. Family support significantly affected the frequency of episodes of homelessness (χ2 (1, *N* = 394) = 20.38, *p* < 0.001, φ = 0.23). The lack of family support increased the chance of living without fixed housing by 3.87 times (odds ratio 95% CI [2.09, 7.18]). More information on these outcomes can be found in **Table 3**.

**Table 3:** Brazilian TGD housing status.

	*n* (%)
How often have you had to move away from your family or friends because you’re transgender? (*n* = 410)	
Never	246 (60.00)
Once or twice	76 (18.54)
Sometimes	42 (10.24)
Many times	46 (11.22)
Have you ever been homeless? (*n* = 422)	
Yes	54 (12.80)
No	368 (87.20)
Thinking about your most recent or current episode of homelessness, where did you sleep or where are you sleeping? (*n* = 54)	
In a shelter	6 (11.11)
In a motel or hotel	18 (33.33)
Outside on the street	12 (22.22)
Outside in parks	4 (7.41)
In a car	18 (33.33)
With a friend or friends	21 (38.88)
With a family member	6 (11.11)
Other	6 (11.11)

### Medical Gender Affirmation Status, Parental Support, Self-Esteem, Willingness to Leave Home and Becoming Homeless

For the SEM analyses, **Table 4** presents the correlation matrix of the various factors included in the model: the gender affirmation procedures, family support, self-esteem, the need to move because of TGD identity and that lack of a home. As can be seen, no variables presented correlation values higher than 0.40. Furthermore, VIF values varied from 1.10 to 1.24. Since there were no correlations equal to or higher than 0.80 and VIF values did not exceed 10.0, there was no indication of multicollinearity and thus SEM models were tested. The SEM results demonstrated adequate fit indices for the model tested (χ2(4) = 7.44, *p* = 0.115; χ2/df = 1.86; CFI = 0.982; TLI = 0.955; RMSEA [90% CI] = 0.047 [<0.001 – 0.099]).

**Table 4:** Correlation matrix among variables included in the structural equation model.

	1. Medical gender affirmation status	2. Had to move from home due to transgender status	3. Self-esteem	4. Family support	5. Homelessness
1. Medical gender affirmation status		-0.021	0.234^∗^	0.183^∗^	0.001
2. Had to move from home due to transgender status			-0.223^∗^	-0.334^∗^	0.332^∗^
3. Self-esteem				0.341^∗^	0.057
4. Family support					-0.184^∗^
5. Homelessness					

*^∗^*p*-value ≤ 0.001.*

**FIGURE 1:**
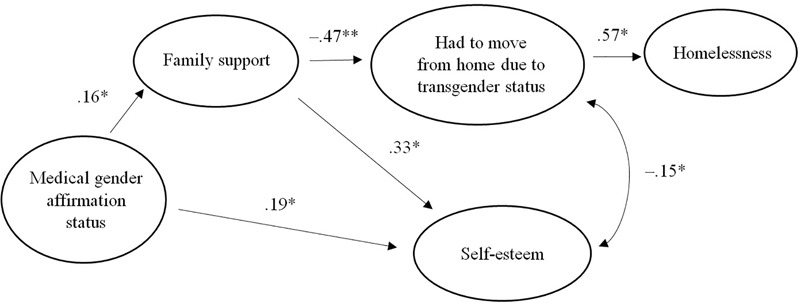
Path model. Medical gender affirmation status, parental support, self-esteem and the willingness to leave home and become homeless. ^∗^*p* < 0.05, ^∗∗^*p* < 0.01, ^∗∗∗^*p* < 0.001.

**Table 5** presents the unstandardized and standardized coefficients and significance levels of the associations in the model. The association between the status of the gender affirmation procedures, family support and self-esteem was significant and indicated that the further TGD individuals advanced in gender affirmation, the more self-esteem and family support they would have. The association between family support and self-esteem indicated that family support was associated with higher self-esteem. Low family support was associated with the willingness to move from home due to one’s TGD status and there was also a significant correlation between low self-esteem and the willingness to move from home due to one’s TGD status. Finally, homelessness was associated with the willingness to move with a large effect size. **Figure 1** depicts the path model among the variables tested in the SEM. An alternative model was tested replacing the correlation between self-esteem and willingness to move from home due to one’s TGD status for a regression path from self-esteem to willingness to move from home. The fit of this alternative model, although acceptable (χ2(4) = 9.51, *p* = 0.049; χ2/df = 2.38; CFI = 0.971; TLI = 0.928; RMSEA [90% CI] = 0.059 [0.003 – 0.109]), was poorer (i.e., significant χ2 and lower CFI) and, most importantly, the theoretical association between these variables reflects a correlation, in which lower self-esteem may lead to willingness to move out, but also having to move from home impacts negatively on one’s self-esteem. Therefore the model with the correlation seems better suited.

**Table 5:** Unstandardized and standardized coefficients and significance levels of the structural equation model.

		Unstandardized coefficient	Standard error	Standardized coefficient	*p*–value
**Path regression estimates**					
Medical gender affirmation status	Family support	0.23	0.095	0.16	0.014
	Self-esteem	1.70	0.491	0.19	0.001
Family support	Self-esteem	2.08	0.407	0.33	<0.001
	Had to move from home due to transgender status	-0.52	0.084	-0.47	<0.001
Had to move from home due to transgender status	Homelessness	0.61	0.108	0.57	<0.001
**Correlation estimate**					
Had to move from home due to transgender status	Self-esteem	-0.86	0.434	-0.15	0.047

In summary, the results suggest the indirect effect of the impact of gender affirmation procedures on homelessness through the effects of family support and the need to move. The indirect effects on homelessness was –0.074 (*p* = 0.024) for gender affirmation procedures and –0.318 (*p* < 0.001). In the model, the combined independent variables accounted for 0.323 of the variance of the homelessness dependent variable.

## Discussion

This study shows the impact of parental support on various aspects of the lives of TGD individuals in a gender affirmation process, mainly self-esteem and housing status. Briefly, this article investigated the association between parental support and risk factors for TGD individuals in different periods of the process of gender affirmation. The lack of parental support increased the chance of living without fixed housing by nearly four times and diminished the TGD individuals’ self-esteem. Both self-esteem and parental support improved during the transition.

The results suggested that parental support was associated with self-esteem. In addition, low family acceptance can be related to the necessity of moving home, and becoming homeless could prevent access to hormonal therapy and sex reassignment surgery, further impairing the self-esteem of TGD individuals.

It is known that closer relationships help TGD individuals build their own identity (Soares et al., 2011). It is clear that parental support affects self-esteem; however, self-esteem may also influence social support. In other words, a positive self-concept could lead to actively developing and maintaining positive social support networks, as well as the perception of having a better social support. Over the course of 4 years, Marshall et al. (2014) evaluated the self-esteem and perceived social support of 961 adolescents. They verified that self-esteem interferes with both their perceptions of social support quality and their social support network size (Marshall et al., 2014). In this vein, knowing that the gender affirmation process enhances self-esteem, and that better self-esteem improves the social support network, the importance of providing hormonal therapy and surgical procedures becomes even more evident (Gorin-Lazard et al., 2013).

Finally, it is noteworthy that the self-esteem score of TGD individuals who received a high level of support did not differ from the self-esteem of the general population (Hutz and Zanon, 2011). Therefore, social support networks could improve the self-esteem of TGD individuals to match that of the general population, even despite the stigma and prejudice that these groups face daily.

Considering the relationship between parental support and housing, results indicated that the lack of parental support increased the chance of living without fixed housing by nearly four times. TGD individuals are more likely to become homeless than cisgender persons (Grant et al., 2011). Homelessness is most likely associated with familial maltreatment, which is higher among LGBT individuals (Choi et al., 2015).

Being homeless was strongly related to survival sex among transgender and gender-non-conforming individuals, indicating that exchanging sex for fundamental resources often is the only way for them to obtain these items of extreme necessity. Furthermore, the results from TransPULSE in Ontario, Canada showed associations between homelessness or under-housing and higher drug use among TGD persons (Scheim et al., 2017). In fact, in contrast with homeless heterosexual youth, homeless LGBTQ youth engage more frequently in survival sex and drug use. They also present more psychopathology and higher numbers of suicide attempts (Cochran et al., 2002).

Currently, most studies have examined all LGBTQ populations, not specifically TGD individuals. Once homeless, TGD individuals still face discrimination and very specific issues in the streets and at shelters. Shelton (2015) investigated the living experiences of TGD persons who experienced homelessness and documented numerous needs that are unique to them, such as the necessity of engaging in medical assistance for gender confirming processes (hormonal therapy and sometimes surgical procedures) and legally changing their names. Other barriers were also reported, especially the constant need to reaffirm and explain their gender identity when facing sex-segregated dormitories and bathrooms, which generates tremendous anxiety and potential alienation.

The results of this study also suggested that the further TGD individuals advance in gender affirmation, the more self-esteem and parental support they will have. In other words, those who have already underwent the procedures had greater parental support than those who planned to complete the process and those who were in doubt or would not complete the process. In this vein, the finding that gender-diverse persons had lower parental support than transgender women and transgender men is consistent with findings in previous studies (Emerson and Rosenfeld, 1996; Lev, 2004), whereas transgender men and transgender women did not differ from each other regarding parental support.

The steps of the gender affirmation procedure closely impacted family support in this study. This aspect can be explained by the changes a family needs to be willing to engage in to redefine their views of gender when facing the procedure (Zamboni, 2006). In addition, the emotional reactions from others may oscillate according to the stage of gender affirmation. The first steps of disclosure tend to be more conflicted, and support can be reduced (Lev, 2004). This fact provides context for why the procedure is negatively associated with family support and for the mediation role of family support on the relationship between the steps of the procedure and the need to move away from home.

This study has some important limitations. First, the study’s cross-sectional design prevents assumptions about causality: it is not possible to determine if social support networks are the reason or the cause of better self-esteem and a more conclusive stage in the medical gender affirmation process. Second, there are important cultural aspects that constrain the results, preventing them from being generalized. For example, in Brazil, violence against TGD individuals is constant and presents itself in numerous ways, from murder and abuse to individual prejudice (Costa et al., 2015). According to the Trans Murder Monitoring Project (Transgender Europe, 2016), Brazil has the highest (absolute) number of homicides of TGD individuals among surveyed countries. Therefore, the participants of this study faced very specific challenges during their lifetime. Third, this sample was predominantly composed of TGD persons with access to the internet and previous contact with TGD-specific communities. Thus, more marginalized individuals may not have had the opportunity to join this study. Finally, although we found good evidences of validity and reliability of the instruments in the protocol, there was no previous study reporting those evidences.

The results presented suggest the necessity of developing interventions for TGD individuals and their families. The field of psychology still lacks studies and validated practices for this population (Blumer and Barbachano, 2008; Benson and Piercy, 2009; Catelan et al., 2017). In addition, clinicians have shown weaknesses in the reception of TGD individuals and their significant others (Blumer et al., 2012), in part explained by the infrequent inclusion of LGBTQ counselor competencies in undergraduate and graduate programs (Biaggio et al., 2003). It is important to consider that TGD individuals have specific issues related to mental health and social support in different moments of gender affirmation. Interventions that promote a better relationship between TGD individuals and their families could prevent TGD vulnerabilities, thus enhancing social support as an important protective factor.

## Author Contributions

AC, BS, EC-S, BdBS, HN, and SK designed the study. AC and DD conceptualized and ran the analysis. BS, AC, BdBS, AF, RC, AB, JS, DD, EC-S, and SK wrote and approved the final version of the manuscript.

## Conflict of Interest Statement

The authors declare that the research was conducted in the absence of any commercial or financial relationships that could be construed as a potential conflict of interest.
