# Corrigendum: The Impact of the Parental Support on Risk Factors in the Process of Gender Affirmation of Transgender and Gender Diverse People

**DOI:** 10.3389/fpsyg.2018.01969

**Published:** 2018-10-12

**Authors:** Bruna L. Seibel, Bruno de Brito Silva, Anna M. V. Fontanari, Ramiro F. Catelan, Ana M. Bercht, Juliana L. Stucky, Diogo A. DeSousa, Elder Cerqueira-Santos, Henrique C. Nardi, Silvia H. Koller, Angelo B. Costa

**Affiliations:** ^1^Departamento de Psicologia, Faculdade Cesuca, Cachoeirinha, Brazil; ^2^Programa de Pós-Graduação em Psicologia, Universidade Federal do Rio Grande do Sul, Porto Alegre, Brazil; ^3^Programa de Identidade de Gênero (PROTIG), Hospital de Clinicas de Porto Alegre (HCPA), Universidade Federal do Rio Grande do Sul, Porto Alegre, Brazil; ^4^Programa de Pós-Graduação em Psicologia, Pontifícia Universidade Católica do Rio Grande do Sul, Porto Alegre, Brazil; ^5^Laboratório de Avaliação e Medição em Psicologia, Universidade Tiradentes, Aracaju, Brazil; ^6^Programa de Pós-Graduação em Psicologia, Universidade Federal de Sergipe, São Cristóvão, Brazil; ^7^Programa de Pós-Graduação em Psicologia Social, Universidade Federal do Rio Grande do Sul, Porto Alegre, Brazil; ^8^Optentia Research Focus Area, North-West University, Vanderbijlpark, South Africa

**Keywords:** parental support, gender affirmation, self-esteem, homelessness, transgender, gender diversity

In the original article, there was a mistake in Table 3 as published. In the original version of the table, total respondents of “have you ever been homeless?” variable was 416 when in fact it is 422. The answer “yes” to that question had originally 4 respondents when in fact it is 54. The corrected Table 3 appears below. The authors apologize for these errors and state that this does not change the scientific conclusions of the article in any way.

The original article has been updated.

Now one can read:

**Table 3:** Brazilian TGD housing status.

	***n* (%)**
How often have you had to move away from your family or friends because you're transgender?(*N* = 410)	
Never	246 (60.00)
Once or twice	76 (18.54)
Sometimes	42 (10.24)
Many times	46 (11.22)
Have you ever been homeless? (*n* = 422)	
Yes	54 (12.80)
No	368 (87.20)
Thinking about your most recent or current episode of homelessness, where did you sleep or where are you sleeping? (*n* = 54)	
In a shelter	6 (11.11)
In a motel or hotel	18 (33.33)
Outside on the street	12 (22.22)
Outside in parks	4 (7.41)
In a car	18 (33.33)
With a friend or friends	21 (38.88)
With a family member	6 (11.11)
Other	6 (11.11)

## Conflict of interest statement

The authors declare that the research was conducted in the absence of any commercial or financial relationships that could be construed as a potential conflict of interest.

