# Corrigendum: The impact of the parental support on risk factors in the process of gender affirmation of transgender and gender diverse people

**DOI:** 10.3389/fpsyg.2023.1206932

**Published:** 2023-05-19

**Authors:** Bruna L. Seibel, Bruno de Brito Silva, Anna M. V. Fontanari, Ramiro F. Catelan, Ana M. Bercht, Juliana L. Stucky, Diogo A. DeSousa, Elder Cerqueira-Santos, Henrique C. Nardi, Silvia H. Koller, Angelo B. Costa

**Affiliations:** ^1^Departamento de Psicologia, Faculdade Cesuca, Cachoeirinha, Brazil; ^2^Programa de Pós-Graduação em Psicologia, Universidade Federal do Rio Grande do Sul, Porto Alegre, Brazil; ^3^Programa de Identidade de Gênero (PROTIG), Hospital de Clinicas de Porto Alegre (HCPA), Universidade Federal do Rio Grande do Sul, Porto Alegre, Brazil; ^4^Programa de Pós-Graduação em Psicologia, Pontifícia Universidade Católica do Rio Grande do Sul, Porto Alegre, Brazil; ^5^Laboratório de Avaliação e Medição em Psicologia, Universidade Tiradentes, Aracaju, Brazil; ^6^Programa de Pós-Graduação em Psicologia, Universidade Federal de Sergipe, São Cristóvão, Brazil; ^7^Programa de Pós-Graduação em Psicologia Social, Universidade Federal do Rio Grande do Sul, Porto Alegre, Brazil; ^8^Optentia Research Focus Area, North-West University, Vanderbijlpark, South Africa

**Keywords:** parental support, gender affirmation, self-esteem, homelessness, transgender, gender diversity

In the published article, there was an error in Table 1 as published. In the section “Gender identity” the rows “Transgender men” and “Transgender women” were reversed. The *n* value for “Transgender men” was given as “265 (61.65)” but should be “124 (29.31).” The *n* value for “Transgender women” was given as “124 (29.31)” but should be “265 (61.65).” The corrected Table 1 appears below.

**Table 1:** Participants sociodemographic data.

	***n* (%)**
Gender identity	
Transgender women	265 (61.65)
Transgender men	124 (29.31)
Gender non-conforming persons	34 (8.04)
Medical gender affirmation status (hormone, surgery, silicone, etc.)	
Done	61 (14.49)
Doing	186 (44.18)
Plan to do	124 (29.45)
Not sure	32 (7.60)
Will not do	18 (4.28)
Age	
18–24	212 (50.72)
25–34	132 (31.58)
35–44	50 (11.96)
45–54	19 (4.55)
55–64	5 (1.20)
Race/color/ethnic	
White	313 (74.00)
Non-white	110 (26.00)
Parda	70 (16.55)
Yellow	12 (2.84)
Indigenous	2 (0.47)
Black	26 (6.15)
Education	
None	6 (1.42)
Fundamental education	40 (9.46)
Middle education	271 (64.07)
Higher education	81 (19.15)
Postgraduate studies	25 (5.91)
Population of city of residence	
5,000–20,000 ihn.	51 (12.06)
20,000–50,000 inh.	38 (8.98)
50,000–1,00,000 inh.	46 (10.87)
1,00,000–5,00,000 inh.	108 (25.53)
More than 5,00,000 inh.	180 (42.55)

The authors apologize for this error and state that this does not change the scientific conclusions of the article in any way. The original article has been updated.

